# Majority of Fortune 500 Companies in 2018 Did Not Recognize Risk of Epidemics Such as COVID-19

**DOI:** 10.3389/fpubh.2021.726987

**Published:** 2021-11-04

**Authors:** Pia D. M. MacDonald, Stacy M. Endres-Dighe, Aaron J. Macoubray, Jamie M. Shorey

**Affiliations:** ^1^RTI International, Research Triangle Park, Durham, NC, United States; ^2^Department of Epidemiology, Gillings School of Global Public Health, University of North Carolina at Chapel Hill, Chapel Hill, NC, United States

**Keywords:** pandemics, epidemics, economics, businesses, emergency preparedness, global health

## Abstract

Infectious disease threats, like the 2002 severe acute respiratory syndrome coronavirus (SARS-CoV) disease, 2009 pandemic influenza A (H1N1), and the current coronavirus disease 2019 (COVID-19), pose multisectoral risk with the potential for wide-ranging socioeconomic disruption. In our globally intertwined economy, the impact of such events can elicit economic shock waves that reach far beyond the country of origin. Review of the 2018 Fortune 500 company 10-K filings shows the majority did not document perceived risks associated with epidemics, outbreaks, or pandemics. Enhanced engagement and investment of the public and private sectors in advancing global health security is needed to effectively prevent, detect, and respond to infectious disease events and ensure U.S. economic security.

## Introduction

The modern world is fueled by globalization with increased interconnectedness and interdependence of countries and people (1). Trade and global economies are interlinked now more than ever. The COVID-19 pandemic has highlighted just how vulnerable our global economy and social fabric are to infectious diseases. As illustrated with the COVID-19 pandemic and the 2014–2016 West Africa Ebola epidemic (2, 3), pathogens move seamlessly among countries, and outbreaks of this magnitude can disrupt trade flows and destabilize global economies. The same was seen in the 2002 SARS-CoV outbreak that had an estimated $11.8–$15 billion US impact on the global economy (4).

The number of annual infectious disease outbreaks worldwide is increasing (5, 6). To mitigate the impacts of this trend, the International Health Regulations (IHR) (2005) require that all countries possess “the ability to detect, assess, report and respond to public health events” (7). In 2015, 67% of countries indicated that they had not yet met the minimum core capacity requirements of IHR 2005 (8). Inadequate capacity to comply with the IHR (2005) remains a persistent challenge (9) that threatens the collective global health security and international health systems, which are only as “strong as their weakest link” (10).

Epidemics, outbreaks, and pandemics pose wide-ranging economic risk with multisectoral impacts that extend far beyond the health sector alone (11). As described previously, the economic consequences of disease events can be significant (3, 12–15), impacting business continuity and resulting in, among other things, supply chain disruptions, trade and travel bans, and long-term employment loss. In 2018, Fortune 500 companies represented two-thirds of the U.S. Gross Domestic Product, $12.8 trillion in revenue, and $21.6 trillion in market value (16). These non-traditional stakeholders in global health security may not recognize the significant impacts of infectious disease threats or be adequately assessing risk to their business.

The Fortune 500 (16) is a list compiled annually which ranks the top 500 publicly and privately held U.S. corporations by total revenue for each respective fiscal year. Corporations are required to submit a Form 10-K annually to the U.S. Securities and Exchange Commission (SEC), with audited annual financial statements and discussions of perceived risks that apply to the company along with analytic results describing the prior year's fiscal operations. The goal of this study was to determine if US Fortune 500 companies were describing perceived risks to their business associated with epidemics, outbreaks, and pandemics in the risk section of their 10-K filings in 2018.

## Materials and Methods

The US SEC Electronic Data Gathering, Analysis, and Retrieval (EDGAR) database (17) provides free access to corporate forms filed with the SEC allowing for public review of a company's financial information. Using the EDGAR database, we obtained the 10-K filing forms for the 2018 Fortune 500 companies. Furthermore, the US SEC EDGAR database categorized Fortune 500 companies into 21 sectors.

Python (18), an open source programming language, was used to scrape, collect, and extract text from the Risk Factors section of each Form 10-K filing located within EDGAR. The software was set to extract the raw html of the SEC filing and identify sections related to risk factors via a set of rules. Code was also developed to search the extracted text for mention of the singular and plural versions of the key terms *pandemic, epidemic*, and *outbreak* in the Risk Factors section. If there was mention, the text surrounding the mention was also collected so that we could manually rule in and out if the context was associated with diseases or pathogens. Mention of key terms was assessed in a dichotomous manner (yes/no mention) by company ranking, revenue and sector. Sector was determined using information from the Fortune 500 (16) website. A Windows files search strategy of the PDF 10-K filings was used to confirm that no companies were overlooked using the Python search program code.

## Results

Of the Fortune 500 companies for 2018, there were Form 10-Ks for 463 of them. Forms for the remaining 37 were not available because of bankruptcy, merger, a lack of public trading, or transition to private ownership.

Differences were noted in the mention of key terms among the 10-K filings available of 2018 Fortune 500 companies. For example, of the 463 Form 10-K filings available, the key term “pandemic” was mentioned at least once by 33% of companies but “outbreak” and “epidemic” were only mentioned by 13 and 15%, respectively (Table 1).

**Table 1:** Distribution of the mention of key terms epidemic, pandemic, and outbreak in the risk factor section of 2018 Fortune 500 companies 10-K filings by sector.

**Sector**	**Companies**		**Pandemic**		**Epidemic**		**Outbreak**		**Outbreak & epidemic**		**Pandemic & outbreak or epidemic**	
	**No.**	**%**	**No.**	**%**	**No.**	**%**	**No.**	**%**	**No.**	**%**	**No.**	**%**
Aerospace & defense	11	2.4	4	0.9	3	0.6	2	0.4	1	0.2	3	0.6
Apparel	5	1.1	2	0.4	3	0.6	1	0.2	0	0.0	1	0.2
Business services	20	4.3	5	1.1	0	0.0	3	0.6	0	0.0	2	0.4
Chemicals	13	2.8	2	0.4	5	1.1	1	0.2	1	0.2	1	0.2
Energy	58	12.5	11	2.4	0	0.0	1	0.2	0	0.0	1	0.2
Engineering & construction	11	2.4	1	0.2	1	0.2	1	0.2	1	0.2	1	0.2
Financials	69	14.9	37	8.0	10	2.2	4	0.9	1	0.2	12	2.6
Food & drug stores	3	0.6	1	0.2	1	0.2	0	0.0	0	0.0	1	0.2
Food, beverages & tobacco	23	5.0	15	3.2	2	0.4	6	1.3	1	0.2	4	0.9
Health care	35	7.6	16	3.5	13	2.8	9	1.9	7	1.5	9	1.9
Hotels, restaurants & leisure	11	2.4	7	1.5	5	1.1	8	1.7	4	0.9	6	1.3
Household products	12	2.6	7	1.5	0	0.0	1	0.2	0	0.0	1	0.2
Industrials	18	3.9	3	0.6	2	0.4	0	0.0	0	0.0	0	0.0
Materials	19	4.1	2	0.4	4	0.9	3	0.6	2	0.4	2	0.4
Media	10	2.2	0	0.0	0	0.0	2	0.4	0	0.0	0	0.0
Motor vehicles & parts	11	2.4	0	0.0	0	0.0	0	0.0	0	0.0	0	0.0
Retailing	44	9.5	15	3.2	8	1.7	6	1.3	2	0.4	9	1.9
Technology	39	8.4	14	3.0	7	1.5	3	0.6	1	0.2	6	1.3
Telecommunications	8	1.7	1	0.2	0	0.0	1	0.2	0	0.0	0	0.0
Transportation	18	3.9	5	1.1	1	0.2	5	1.1	0	0.0	2	0.4
Wholesalers	25	5.4	6	1.3	3	0.6	5	1.1	3	0.6	2	0.4
**Total (%)**	**463**	**100**	**154**	**33.1**	**68**	**14.5**	**62**	**13.1**	**24**	**5.0**	**63**	**13.3**

Differences in mention exist by company sector (Table 1). Among all 21 US SEC EDGAR-identified sectors, key terms were mentioned as potential risks most often in the 10-K filings of the Financial (*N* = 51), Health Care (*N* = 38), and Retailing (*N* = 29) companies and least often among Food and Drug Stores (*N* = 2), Media (*N* = 2), Telecommunications (*N* = 2), and Motor Vehicles & Parts (*N* = 0). “Pandemic” was mentioned as a risk factor most often among Financial (37) and Health care (16) sectors. Neither “pandemic” nor “epidemic” were identified as risk factors in the Media or Motor Vehicles & Parts sectors 10-K filings.

**Figure 1:**
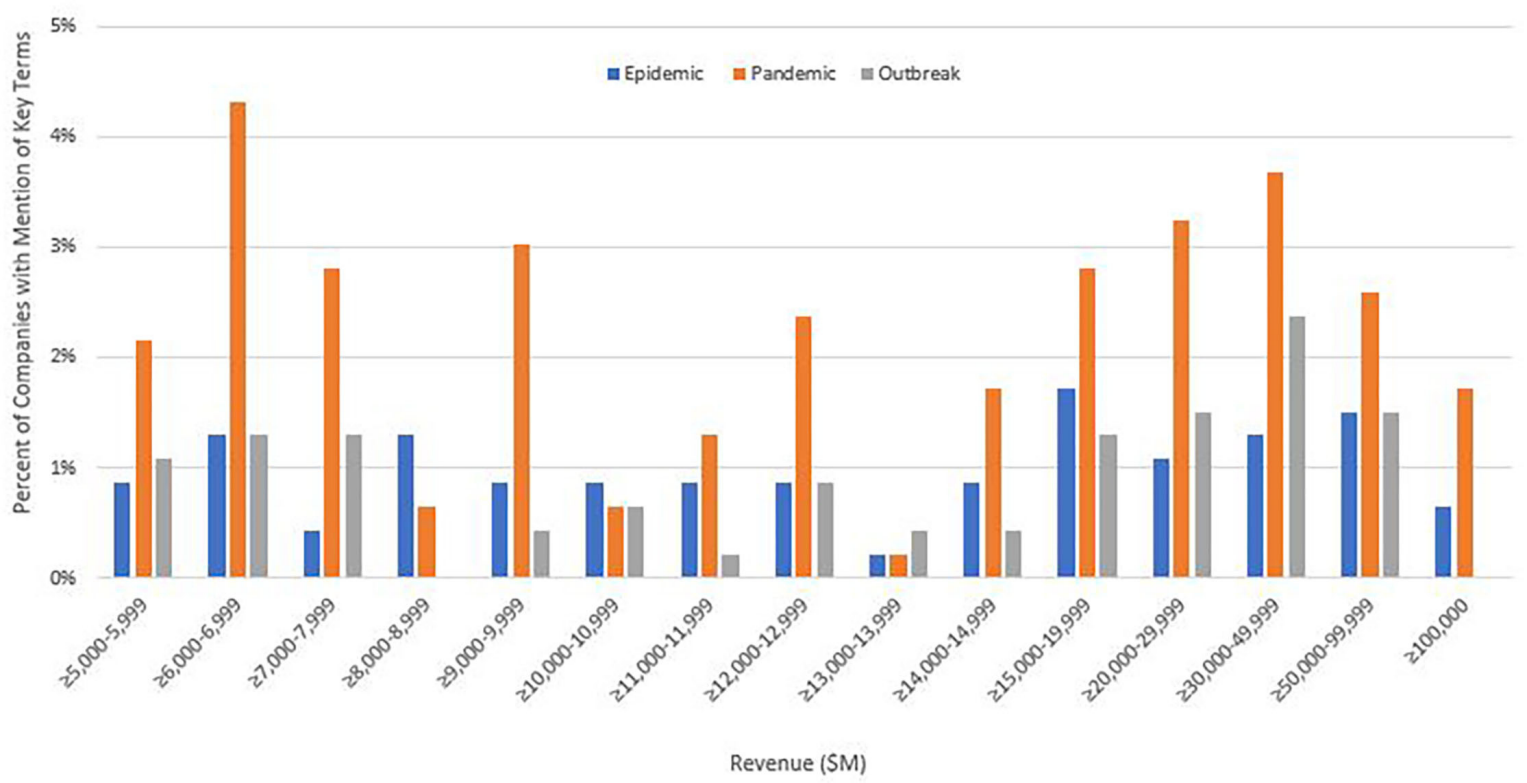
Percent of 2018 Fortune 500 companies with mention of epidemic, pandemic, and outbreak in the risk factor section of their 10-K filings by revenue. ≥5,000–5,999 (*N* = 31), ≥6,000–6,999 (*N* = 59), ≥7,000–7,999 (*N* = 46), ≥8,000–8,999 (*N* = 23), ≥9,000–9,999 (*N* = 29), ≥l0,000–10,999 (*N* = 21). ≥11000–11,999 (*N* = 19), ≥12,000–12,999 (*N* = 30), ≥13,000–13,999 (*N* = 11), ≥14,000–14,999 (*N* = 21), ≥15000–19,999 (*N* = 36), ≥20,000–29,999 (*N* = 50), ≥30,000–49,999 (*N* = 32), ≥50,000–99,999 (*N* = 31), ≥100,000 (*N* = 24).

Moreover, differences in mention exist by company ranking (Figure 1). Overall, companies with the largest total revenue identified one of the three key terms as a potential risk more often than lower ranking corporations. Over 4% of all companies ranging in revenue ≥$6,000–$6,999 M mentioned *pandemic*, followed closely by ≥$30,000–$49,999 M at 3.7% and ≥$20,000–$29,999 M at 3.2%. At 1.7 and 1.5%, respectively, companies ranging in revenue ≥$15,000–$19,999 M and ≥$50,000–$99,999 M mentioned *epidemic* most often. *Outbreak* was mentioned by 2.4% of companies ranging in revenue ≥$30,000–$49,999 M, and 1.5% by those valued at ≥$20,000–$29,999 M and ≥$50,000–$99,999 M. Interestingly, none of the companies ranging in revenue ≥$8,000–$8,999 or ≥$100,000 M had any mention of outbreak.

## Discussion

Our findings demonstrate that overall, the majority of Fortune 500 companies are not describing perceived risks to their business associated with pandemics, epidemics, or outbreaks. Of the corporations considering the economic costs of disease events, those with the highest total revenue tended to mention one of the key terms more often than their lower ranking counterparts. This trend indicates that corporations in the top revenue percentiles may be more aware of how, in our globally intertwined economy and supply chains, local and global health emergencies can pose significant direct and indirect risk for US business continuity.

Of the three key terms we assessed, *pandemic* was mentioned most often as a risk factor in the 10-K filings. There are several possible explanations for this. First, unlike *outbreak* or *epidemic*, which could be viewed by companies as localized, a pandemic threatens to spread worldwide. The mobility of a pandemic instills greater fear of its potential “reach” and may therefore result in heightened recognition by US companies as a potential risk factor. A second possible explanation has to do with the severity of pandemic disease events and associated economic cost. Typically, the poorest countries, those without basic primary health infrastructure and infection control mechanisms, suffer the most (10). Inadequate sanitation and the lack of clean water or basic health services act as disease amplifiers, complicating outbreak preparedness and response and enabling an infectious pathogen to spread quickly (10). Aside from the significant loss of life, pandemics can devastate already fragile markets. As demonstrated by the 2003 SARS (12) and 2009 H1N1 influenza pandemics (10), the impact of widespread devastation on low-resource countries reverberates throughout the entire global community and can create economic instability in the United States (14, 15). Considering the potential impact, it is not surprising that Fortune 500 companies are readily identifying pandemics as a risk to business continuity.

Among the 21 sectors, Financials, Health Care, and Retailing mentioned the terms most often. Interestingly, Motor Parts & Vehicles failed to acknowledge pandemic, outbreak, or epidemic as a risk factor in their 10-K filings. These findings suggest that, unlike other sectors, US Motor Parts & Vehicles corporations do not view these events as posing a significant risk to their company or its securities. The automotive industry is a global one with most parts manufactured outside of the United States. In 2018, US companies purchased $71.5 billion worth of imported auto parts and over 50% were supplied by China and Mexico (19). Limited manufacturer sourcing can be inherently risky with supply disruptions, such as those resulting from a disease outbreak, causing rippling economic impacts. The COVID-19 outbreak originating in China has highlighted the extent to which a disease event can impact the automotive industry (20, 21). Numerous auto parts plants, including those run by Hyundai, Tesla, Ford, and Nissan were shut down because of the outbreak (20). Hyundai, the fifth largest automaker in the world, suspended production in a South Korean factories because of shortages of Chinese parts (20), most of which did not become operational until February 14, 2020 (22).

Food & Drug Stores failed to mention “outbreak” as a potential economic risk factor in their 10-K filings. Even if they do not move from one country to another, outbreaks can disrupt trade by destabilizing foreign economies that serve as export markets (15). Considering 80% of active ingredients in America's pharmaceutical and over-the-counter drugs are imported from China and India (23), it is likely that these corporations have grossly underestimated the potential impacts a foreign outbreak can have on the global supply chain. Industries worldwide rely on China for its efficient factories. If we have learned nothing else from previous and current outbreaks, it is that disease events can disrupt production and the disruption of manufacturers is felt throughout the global supply chain (1, 3, 14, 20, 21). Food & Drug Stores are not immune to the economic impact of outbreaks and, in fact, their risk is likely to increase with the launch of the Chinese governments “Made in China 2025” industry plan (24). According to this policy, China seeks to become the world's pharmacy by focusing on the promotion of biomedicine and high-end medical equipment manufacturing and increasing medical exports. The economic impact of an outbreak on the US Food & Drug Stores sector will increase proportionally as the United States becomes increasingly reliant on one country as the sole source of vital medicines.

This study had several limitations. First, our assessment relied on any mention of the three key terms and did not examine the 10-K filings beyond presence or absence of the terms. For example, the quality of the risk described in the filing was not assessed. Second, we could not assess the extent to which companies may have addressed risks associated with disease related events using different terminology than our three key terms. One could assume, for example, that corporations in the health sector would be more familiar with public health vocabulary such as *pandemic, outbreak*, and *epidemic* and thus use these terms more readily in their 10-K filings when describing risk than their non-health sector counterparts.

Although this study did not investigate whether Fortune 500 companies mentioned risks associated with natural and man-made disasters, the impact of those events on supply chain disruption should also be considered. For example, in September 2018, Hurricane Maria devastated Puerto Rico. The category 4 hurricane severely damaged Baxter International manufacturing plants, a key manufacturer of IV bags, initiating what would become a major national shortage (25). Six months after the hurricane, the scarcity of the IV bags reached crisis level, illustrating the rippling effects damage to a supply chain in one part of the world can have on the system as a whole. No matter the market, it is necessary to establish stronger supply chain safety margins to stabilize and defend American economies during emergencies.

## Conclusions

All sectors are potentially susceptible to the impacts of pandemics, epidemics, and outbreaks, yet most Fortune 500 companies are not describing perceived risks to their business. The private sector is largely under-recognized and represented in initiatives to enhance the health security of the United States and other nations.

For US business interests to remain secure and stable, health security is paramount. The Global Health Security Agenda (26) offers a framework to enhance capacity globally and ensure compliance with the International Health Regulations. An all-of-society approach to health security is needed; the private sector's engagement in global health security is vital for our collective ability to effectively prevent, detect, and respond to infectious disease events.

## Data Availability Statement

The raw data supporting the conclusions of this article will be made available by the authors, without undue reservation.

## Author Contributions

AM: review and editing. JS: data acquisition. All authors contributed to the article and approved the submitted version.

## Funding

This study was funded by RTI International.

## Conflict of Interest

The authors declare that the research was conducted in the absence of any commercial or financial relationships that could be construed as a potential conflict of interest.

## Publisher's Note

All claims expressed in this article are solely those of the authors and do not necessarily represent those of their affiliated organizations, or those of the publisher, the editors and the reviewers. Any product that may be evaluated in this article, or claim that may be made by its manufacturer, is not guaranteed or endorsed by the publisher.
